# Association Between BMI and Tumour Regression After Neoadjuvant Therapy in Oesophageal Cancers: Insights From a German Nationwide Registry

**DOI:** 10.1002/cam4.72169

**Published:** 2026-09-18

**Authors:** Susanne D. Otto, Ioannis Pozios, Wael Rayya, Christian H. W. Schineis, Swantje Malinka, Rahel M. Strobel, Jonas Staudacher, Christoph Treese, Tim Vilz, Pia Jäger

**Affiliations:** ^1^ Department of General and Visceral Surgery Charité – Universitätsmedizin Berlin, Corporate Member of Freie Universität Berlin und Humboldt‐Universität zu Berlin Berlin Germany; ^2^ Department of Gastroenterology, Infectiology and Rheumatology Charité – Universitätsmedizin Berlin, Corporate Member of Freie Universität Berlin und Humboldt‐Universität zu Berlin Berlin Germany; ^3^ Berlin Institute of Health at Charité – Universitätsmedizin Berlin Berlin Germany

**Keywords:** AEG, Becker grade, BMI, neoadjuvant therapy, obesity, oesophageal cancer, tumour regression

## Abstract

**Background:**

For locally advanced oesophageal carcinoma, multimodal therapy is the standard of care, but prognosis remains poor. Obesity has risen markedly in the last decades. The impact of obesity on response to neoadjuvant treatment remains unclear. This study evaluates the association between body mass index (BMI) and histopathological tumour regression following neoadjuvant therapy in oesophageal cancer.

**Materials and Methods:**

We performed an analysis using the prospective database from the DGAV|registry oesophageal surgery provided by the German Society for General and Visceral Surgery (Deutsche Gesellschaft für Allgemein‐ und Viszeralchirurgie, DGAV). The primary outcome was histopathological tumour regression grade by the Becker classification. The primary explanatory variable was BMI. Descriptive statistics were followed by uni‐ and multivariate regression analyses adjusted for tumour histology, neoadjuvant regimen, age, sex, ECOG status, comorbidities, dysphagia and weight loss. Associations were evaluated using a multivariate regression (partial proportional odds) model.

**Results:**

Six hundred and forty‐seven patients were included, comprising 432 adenocarcinomas (including AEG I/II) and 215 squamous cell carcinomas (SCC). After adjustment for clinical and tumour‐related covariates, higher BMI was independently associated with a favourable histopathological response: No association was observed for the transition to Becker grade ≥ 1b; however, higher BMI was associated with reduced odds of Becker grade ≥ 2 (OR 0.97, 95% CI 0.94–1.00, *p* = 0.04) and Becker grade ≥ 3 (OR 0.93, 95% CI 0.90–0.96, *p* < 0.001). In addition, SCC, absence of dysphagia, radiochemotherapy and low ECOG score were independently associated with favourable regression, whereas no independent associations were observed for age, sex, diabetes status, comorbidities or pretherapeutic weight loss.

**Conclusion:**

Analysis of the German DGAV|registry revealed that higher BMI was significantly associated with improved histopathological tumour regression following multimodal therapy for oesophageal cancer. These findings suggest potential differences in tumour biology among patients with elevated BMI, underscoring the need for further mechanistic investigation.

**Abbreviations**
AEG: adenocarcinoma of the oesophagogastric junction
AIC/BIC: Akaike and Bayesian information criteria
ANOVA: analysis of variance
ASA: American Society of Anesthesiologists Physical Status Classification
BMI: body mass index (kg/m^2^)
CCI: Charlson comorbidity index
CI: confidence interval
CROSS: chemoradiotherapy for oesophageal tumours followed by surgery
CTX: chemotherapy
DGAV: Deutsche Gesellschaft für Allgemein‐ und Viszeralchirurgie (German Society for General and Visceral Surgery)
ECF/ECX: epirubicin, cisplatin, fluorouracil/capecitabin
ECOG: Eastern Cooperative Oncology Group
FLO: fluorouracil, leucovorin, oxaliplatin
FLOT: fluorouracil, leucovorin, oxaliplatin, docetaxel
GERD: gastro‐oesophageal reflux disease
IGF‐1: insulin‐like growth factor 1
N.A.: not available/not applicable
NYHA: New York Heart Association
OR: odds ratio
RCTX: radiotherapy
SCC: squamous cell carcinoma
TRG: tumour regression grade (Becker score)
VIF: variance inflation factor
WHO: World Health Organization

## 1 Introduction

### 1.1 Epidemiology of Oesophageal Cancer

The incidence of oesophageal cancer has increased in recent decades, making it the eleventh most prevalent cancer and the seventh leading cause of cancer‐related death worldwide, with an estimated 511,000 new cases and 445,000 deaths in 2022 [1, 2]. The prognosis is poor with an average 5‐year survival rate of only 15%–25%, mainly due to the aggressive nature and the often late onset of significant symptoms, resulting in a high rate of advanced tumour stages at the time of first diagnosis [3]. Histologically, oesophageal squamous cell carcinoma (SCC) is the dominant subtype worldwide, especially in Asia, Eastern Africa and South America. However, in Europe and North America an increase particularly of oesophageal adenocarcinoma and adenocarcinoma of the oesophagogastric junction (AEG) has been observed, making it the dominant subtype in these regions [4]. Main risk factors associated with SCC are tobacco and alcohol consumption, achalasia, caustic injuries and hot beverages, whereas adenocarcinomas are mainly associated with gastroesophageal reflux disease (GERD), Barrett's oesophagus, abdominal obesity and tobacco use [3].

### 1.2 Implication of Obesity

Worldwide, the prevalence of obesity has more than doubled since 1990, and an estimated 16% of the global adult population is now living with obesity. In 2022, according to the definition of the World Health Organization (WHO), an estimated 2.5 billion adults were overweight with a body mass index (BMI) of ≥ 25.0 kg/m^2^. Of these, an estimated 890 million were obese with a BMI ≥ 30.0 kg/m^2^ [5], making overweight a relevant health issue worldwide.

Obesity is widely recognized as a major risk factor for cancer development and is linked to increased incidence and mortality in multiple cancers, including colon, pancreatic and postmenopausal breast cancer [6, 7]. In oesophageal cancer, higher body weight is particularly associated with an increased risk of adenocarcinomas, including AEG, up to 40% of which are linked to obesity [8, 9]. Pathophysiologically, obesity contributes to elevated intra‐abdominal pressures, increased transient relaxations of the lower oesophageal sphincter and a higher prevalence of hiatus hernia. These mechanisms promote the development of GERD and its potential dysplastic progression to Barret's oesophagus [8, 9]. Barrett's oesophagus, which is seen in about 10%–16% of patients with severe and long‐standing GERD, is one of the strongest risk factors for oesophageal adenocarcinoma and AEG [8]. Independent of GERD, obesity leads to low‐grade chronic inflammation and systemic metabolic alterations, which are considered part of the broader mechanisms driving carcinogenesis [8].

### 1.3 Treatment Approaches

Oesophageal cancer is an aggressive cancer and remains challenging to treat. The case‐fatality ratio is 83%, substantially higher than that of many other common malignancies, such as colon or breast cancer [8]. However, the prognosis for patients treated with curative intent has improved, particularly since multimodal treatment became the standard of care for locally advanced resectable disease, resulting in a 5‐year overall survival rate of 47% in the landmark CROSS trial [10]. According to the current German S3 guideline ‘Oesophageal carcinomas’, perioperative chemotherapy or preoperative chemoradiotherapy is recommended for locally advanced resectable adenocarcinoma (including Type I/II AEG), while preoperative chemoradiotherapy is recommended for locally advanced resectable SCC [11]. Furthermore, recent developments in immunotherapy and target therapies are gaining increasing attention [4].

Despite the proven benefit of neoadjuvant treatment in down‐staging oesophageal cancer, therapeutic responses vary widely. A lack of response to neoadjuvant treatment is associated with a poor overall outcome, underscoring the importance of identifying prognostic factors. Known factors which affect pathologic complete response include age, clinical stage, histological subtype, tumour size and tumour differentiation [12, 13].

### 1.4 Aim

There is limited knowledge about the impact of obesity on treatment strategies and outcomes. Given the rising global prevalence of obesity, this represents a pressing clinical concern. Moreover, a deeper understanding may help identify therapeutic targets or strategies that extend beyond this specific patient group and provide broader insights into tumour biological response patterns.

Therefore, the aim of this study is to provide insight into the response to neoadjuvant treatment in oesophageal cancer, with a particular focus on BMI as a potential modulator of treatment response.

## 2 Methods

### 2.1 Data Basis and Included Variables

We performed an analysis using the nationwide multi‐centre prospective database from the German DGAV|registry oesophageal surgery provided by the German Society for General and Visceral Surgery (Deutsche Gesellschaft für Allgemein‐ und Viszeralchirurgie, DGAV). Participation in the DGAV|registry is not mandatory, but strongly recommended for treatment centres nationwide. All patients have given informed consent to the collection and analysis of their medical data and history prior to the data collection. Data analysis was performed using selected variables from the pseudonymized data export of the DGAV|registry. The analysis included all patients who underwent surgery after neoadjuvant treatment for oesophageal cancer, including AEG Siewert types I and II, between 2021 and 2024. The type of therapy administered is described in detail below. No patients who met the inclusion criteria were excluded.

The main outcome parameter was the histopathological regression grade by the Becker classification, determined from the resected specimen by histopathological assessment. It distinguishes between grade 1a—complete response (no tumour cells), grade 1b—subtotal response (< 10% tumour cells), grade 2—partial response (10%–50% tumour cells) and grade 3—minimal or no response (more than 50% tumour cells) [11]. This variable was treated as an ordinal scaled factor.

The primary explanatory variable was the BMI in kg/m^2^ according to WHO definitions [14]: assessed shortly before or at the time of surgery. BMI was treated as a continuous variable.

Variables extracted from the DGAV|registry were recoded and harmonized as required to enable statistical analysis. Comorbidities were captured using a composite variable to ensure model stability, given the assessment of multiple conditions, including cerebrovascular disease, coronary artery disease, New York Heart Association (NYHA) class, dialysis dependency, chronic obstructive pulmonary disease, peripheral arterial occlusive disease and Child–Pugh score. The composite variable indicated the presence of at least one of these comorbidities. Diabetes mellitus was considered separately and was not included in the composite variable, in line with the specific focus of the present study. To simplify the ECOG (Eastern Cooperative Oncology Group) performance variable and thereby improve statistical stability, a simplified ECOG variable was generated, distinguishing ECOG binarily between ECOG < 2 and ECOG ≥ 2 as ordered factors.

Tumour entities were classified based on anatomical location and histological subtype. In line with current guideline recommendations [11, 15], AEG classified as AEG type III (tumour centre > 2 cm distal to the cardia, according to the Siewert classification) are no longer considered oesophageal cancers and were therefore not included in the analysis. All other tumours were categorized as adenocarcinoma or SCC according to WHO histology. For patients with nonclassifiable tumours in the surgical specimen, histopathological reports from pre‐neoadjuvant biopsies were used. Adenocarcinoma subtypes were pooled for analysis. This classification was used consistently across all subgroup analyses.

Neoadjuvant treatment protocols, reflecting standard clinical practice and guideline recommendations for perioperative treatment of gastro‐oesophageal cancers in Germany, were categorized based on the combination of preoperative chemotherapy and radiotherapy. For simplification and to improve the robustness of the statistical models, patients were summarized into the two groups: chemotherapy (CTX) and radiochemotherapy (RCTX). Patients classified into the CTX group were receiving either triple‐agent chemotherapy (with or without taxane) without concurrent radiotherapy (e.g., FLOT, ECF/ECX) or double‐agent chemotherapy without radiotherapy (e.g., FLO). Patients assigned to the RCTX category underwent double‐agent chemotherapy or triple‐agent chemotherapy (including a taxane) in combination with radiotherapy (e.g., CROSS). Patients with missing data were classified as not applicable (N.A.). Categorization was cross‐checked against raw data and found to be consistent with treatment documentation. This variable was used in all analyses involving neoadjuvant therapy stratification.

### 2.2 Descriptive Statistics

Statistical analyses were performed using R version 4.4.2 [16], with descriptive statistics and data visualization conducted within the tidyverse framework [17] using ggplot2 for graphical representation [18]; statistical significance was defined as *α* ≤ 0.05.

To characterize patients' clinical condition and overall health status, ASA (American Society of Anaesthesiologists Physical Status Classification), CCI (Charlson comorbidity index) and ECOG classifications were applied [19, 20], and comorbidities were assessed accordingly. Patient‐specific clinical characteristics are reported, including sex, age, ECOG performance status, dysphagia, weight loss and comorbidity burden, as well as histological tumour type, tumour localization, surgical procedure and neoadjuvant treatment regimen.

### 2.3 Univariate Statistics

Data were described using contingency tables for categorical variables and summary measures for continuous variables. Univariate analyses were conducted in R [16] to assess associations between BMI and histopathological regression. Parametric analysis of variance (ANOVA) was initially applied; however, as distributional assumptions were violated based on Shapiro–Wilk testing and graphical assessment, analyses were subsequently performed using non‐parametric methods, including Kruskal–Wallis tests with Bonferroni‐adjusted post hoc comparisons and pairwise Wilcoxon rank‐sum tests, implemented using standard R functionality and supporting statistical packages [21, 22]. All analyses were repeated with and without inclusion of underweight patients as a sensitivity analysis.

### 2.4 Multivariate Statistics

After univariable analyses, a multivariable ordinal regression model was constructed. BMI was included as the primary explanatory variable of interest irrespective of its univariable significance, as the univariable analyses were not used as the sole criterion for variable selection and unadjusted associations may be confounded or masked by clinically relevant covariables. Covariables were predefined based on clinical evidence and established predictors in the existing literature [23, 24, 25]. Specifically, we adjusted for sex, age, ECOG performance status, dysphagia, weight loss, comorbidity burden, histological tumour type, tumour localization, surgical procedure and neoadjuvant treatment regimen. These covariables were included as permitted by model stability.

Model specification was guided by variable characteristics, model stability and avoidance of overfitting. An ordinal regression approach was selected, as it best reflects the properties of the Becker regression score. The required ordinal scaling of the outcome variable and independence of observations were given by study design. Multicollinearity was assessed using variance inflation factors (VIFs) calculated from an equivalent multivariable linear model. Variable properties and the proportional odds (equal slopes) assumption were evaluated for each covariate, and the model was adapted accordingly, allowing non‐parallel effects for variables violating this assumption. Model fit was assessed using AIC and BIC, as well as McFadden's pseudo‐*R* ^2^, which was calculated from the ratio of the log‐likelihoods of the final model and the intercept‐only (zero‐) model. In the presence of potential interaction or multicollinearity, further sensitivity or interaction analyses were performed, as indicated by variance inflation factors (VIF) exceeding 2.

## 3 Results

### 3.1 Data Basis

The database comprises DGAV|registry data from all patients with oesophageal cancer, including AEG I/II, who underwent neoadjuvant therapy followed by surgery at participating hospitals between 2021 and 2024 and for whom complete histological data were available.

### 3.2 Descriptive Statistics

The study cohort comprises *n* = 647 patients, of whom *n* = 432 (66.77%) are classified as adenocarcinoma (including AEG I and II) and *n* = 215 (33.23%) as squamous cell carcinoma. The vast majority (98.76%) of these patients underwent elective surgery, while eight patients (1.24%) received emergency surgery. The characteristics of the patients included are presented in Table 1. Tumour characteristics based on the histopathological stage upon resection are given in Table 2.

**TABLE 1:** Overall characteristics of all patients included (*n* = 647; absolute numbers [*n*], ratio of the respective entity [%]).

		Adenocarcinoma		SCC	
		*n*	%	*n*	%
Overall		432	100.00	215	100.00
Sex	Male	366	84.72	145	67.44
	Female	66	15.28	70	32.56
ECOG status	0	210	48.61	84	39.07
	1	183	42.36	97	45.12
	2	36	8.33	32	14.88
	3	3	0.69	2	0.93
ASA classification	1	28	6.48	2	0.93
	2	195	45.14	85	39.53
	3	204	47.22	124	57.67
	4	5	1.16	4	1.86
Dysphagia	Yes	317	73.38	168	78.14
	No	115	26.62	47	21.86
Weight loss	Yes	128	29.63	74	34.42
	No	304	70.37	141	65.58
Alcohol abuse	Yes	40	9.26	38	17.67
	No	392	90.74	177	82.33
Elective surgery	Yes	428	99.07	211	98.14
	No	4	0.93	4	1.86
Diabetes mellitus		73	16.9	23	10.7
Cerebrovascular		21	4.86	12	5.58
Coronary artery disease		65	15.05	31	14.42
Heart failure		74	17.13	42	19.53
Dialysis		0	0.0	1	0.47
COPD		28	6.48	29	13.49
Peripheral arterial disease		5	1.16	6	2.79
Liver cirrhosis		13	3.01	8	3.72
		**Mean**	**SD**	**Mean**	**SD**
Age (years)		63.54	9.85	64.90	8.91
BMI (kg/m^2^)		26.85	4.78	23.44	4.35
CCI index		2.62	0.99	2.62	0.87
Smoking cessation, years before surgery		6.26	8.99	5.90	12.11
Pack years		9.84	18.01	15.88	22.18

**TABLE 2:** Tumour characteristics upon resection of all patients included (*n* = 647; absolute numbers [*n*], ratio of the respective entity [%], median, interquartile range IQR 25%).

	Adenocarcinoma		SCC	
	*n*	%	*n*	%
Localization				
Overall	432	100.00	215	100.00
Cervical	1	0.23	8	3.72
Intrathoracic, with tracheobronchial relation	6	1.39	13	6.05
Intrathoracic, no tracheobronchial relation	187	43.29	161	74.88
Kardia (1 cm proximal to 2 cm distal to Z‐line)	238	55.09	33	15.35
Pathological T‐classification				
Overall	432	100.00	215	100.00
T0	86	19.91	82	38.14
T1a (lamina propria/muscularis mucosae)	25	5.79	9	4.19
T1b (submucosa)	42	9.72	20	9.30
T2 (muscularis propria)	72	16.67	38	17.67
T3 (subserosa)	195	45.14	62	28.84
T4a (serosal perforation)	10	2.31	1	0.47
T4b (adjacent structures)	1	0.23	1	0.47
Tx (unclassifiable)	1	0.23	2	0.93
Pathological M‐status				
M0	416	96.30	211	98.14
M1	16	3.70	4	1.86
Pathological N‐status				
N0 (no infiltrated lymph nodes)	218	50.46	135	62.79
N+ (≥ 1 infiltrated lymph node)	214	49.54	80	37.21
	**Median**	**IQR**	**Median**	**IQR**
Lymph nodes information				
Lymph nodes examined	29	15	26	12
Lymph nodes infiltrated	0	3	0	1

Neoadjuvant therapy was administered in accordance with prevailing guideline recommendations. Since these recommendations changed during the data collection period, some treatment regimens that are no longer considered standard of care were included. Allocation to oncological protocols followed the approach described in Section 2. Data on the number of chemotherapy cycles and the total radiation dose (Gray) were not available. However, information was available on whether the recommended neoadjuvant therapy was completed as planned. Information about the neoadjuvant therapy schemes is given in Tables 3 and 4.

**TABLE 3:** Administered neoadjuvant therapy schemes (*n* = 647; absolute numbers [*n*], ratio of the respective entity [%]).

	Adenocarcinoma		SCC	
	*n*	%	*n*	%
Protocol				
Overall	432	100.00	215	100.00
Chemotherapy	342	79.17	30	13.40
Radiochemotherapy	79	18.76	169	84.92
N.A.	11	2.55	16	7.44
Protocol, detailed				
Overall	432	100.00	215	100.00
Triplet, including taxane (e.g., FLOT)	294	68.06	23	10.70
Triplet, without taxane (e.g., ECF/ECX)	34	7.87	1	0.47
Doublet (e.g., FLO)	14	3.24	6	2.79
Radiochemotherapy, double agent (e.g., CROSS)	57	13.19	129	60.00
Radiochemotherapy, triple agent	22	5.09	40	18.60
N.A.	11	2.55	16	7.44
Completion status				
Overall	432	100.00	215	100.00
Completed as planned	397	91.90	185	86.05
Terminated due to side effects	17	3.94	13	6.05
Terminated due to tumour progression	3	0.69	0	0.00
Terminated for other reasons	2	0.46	3	1.40
Patient refused continuation	1	0.23	0	0.00
N.A.	8	1.85	11	5.12
Unknown	4	0.93	3	1.40

**TABLE 4:** Administered radiotherapy (*n* = 647; absolute numbers [*n*], ratio of the respective entity [%]).

	Adenocarcinoma		SCC	
	*n*	%	*n*	%
Indication/reason				
Yes, within clinical trail	11	2.55	4	1.86
Yes, according to recommendation	74	17.13	176	81.86
No, no recommendation	344	79.63	34	15.81
No, refused by patient	1	0.23	0	0.0
No, patient comorbidities	2	0.46	0	0.0
No, tumour stenosis	0	0.0	1	0.47
Radiotherapy dose				
< 30 Gy	1	0.23	3	1.40
30 Gy	1	0.23	5	2.33
35 Gy	0	0	4	1.86
40 Gy	57	13.19	121	56.28
45 Gy	20	4.63	27	12.56
50 Gy	5	1.16	10	4.65
55 Gy	0	0.0	1	0.47
60 Gy	0	0.0	4	1.86
> 60 Gy	1	0.23	5	2.33
N.A.	347	80.32	35	16.28
Completion status				
Completed as planned	82	18.98	169	78.60
Terminated due to side effects	1	0.23	4	1.86
Terminated for other reasons	0	0.0	2	0.93
N.A.	347	80.32	35	16.28
Unknown	2	0.46	5	2.33
Radiotherapy recommendation				
Yes	301	69.68	200	93.02
No	38	8.80	7	3.26
N.A.	93	21.53	8	3.72

**FIGURE 1:**
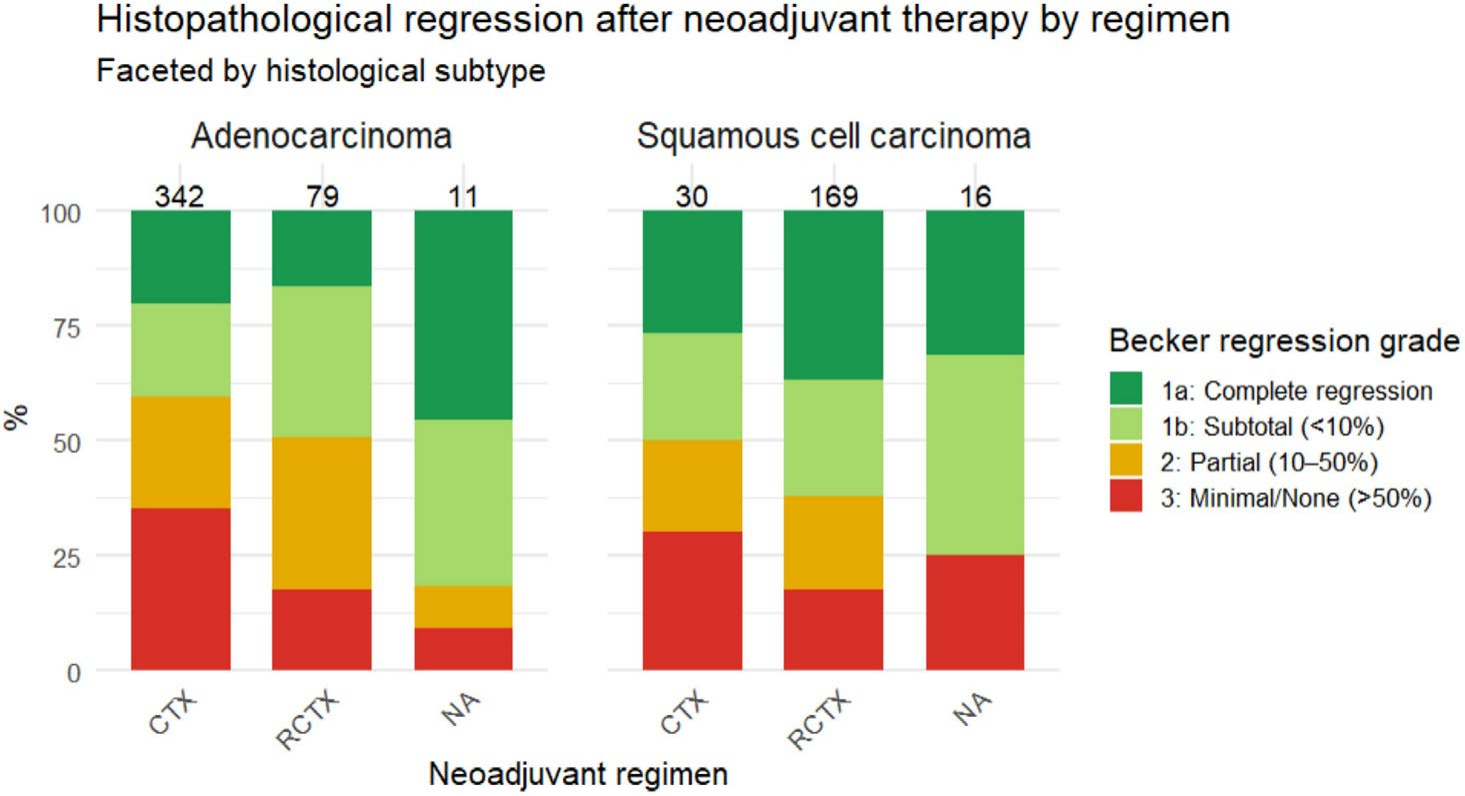
Neoadjuvant therapeutic regimens administered to patients with oesophageal cancer. A visual trend suggests that higher regression grades (more 1a and 1b responses) occur more frequently in SCC compared to adenocarcinoma.

**FIGURE 2:**
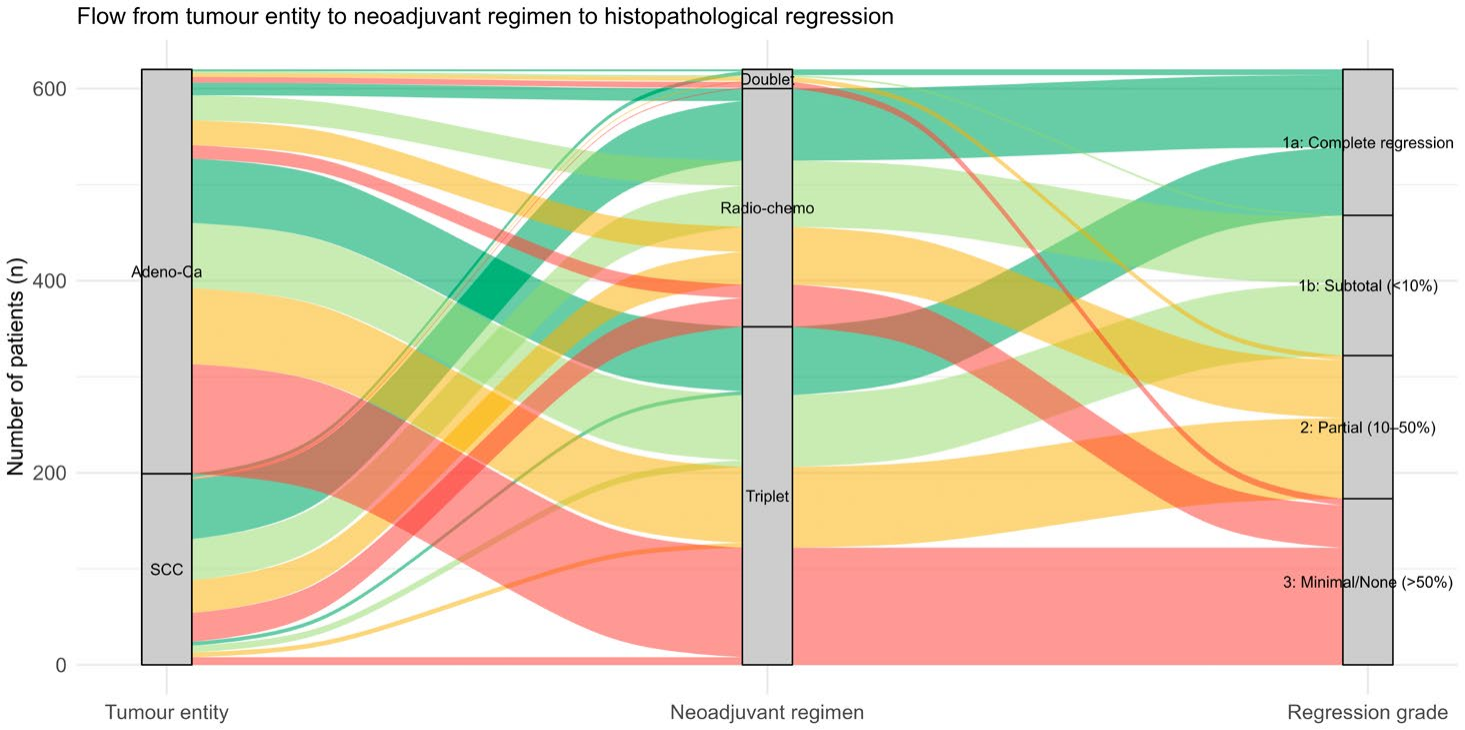
Alluvial plot illustrating the flow from tumour entity to administered neoadjuvant therapy and resulting histopathological regression grades.

Histopathological regression was assessed on surgical resection specimens and was available for all *n* = 647 patients with oesophageal cancer, including *n* = 432 patients with adenocarcinoma and *n* = 215 patients with SCC. Complete tumour regression, defined as the absence of viable tumour cells (grade 1a), was observed in 20.14% of adenocarcinoma patients (*n* = 87) and in 34.88% of squamous cell carcinoma patients (*n* = 75). Subtotal regression with < 10% residual tumour (grade 1b) was found in 23.15% of adenocarcinoma patients (*n* = 100) and 26.51% of SCC (*n* = 57). Partial regression with 10%–50% residual tumour (grade 2) occurred in 25.46% of adenocarcinoma cases (*n* = 110) and 18.60% of SCC cases (*n* = 40). Minimal or no regression with more than 50% residual tumour (grade 3) was reported in 31.25% of adenocarcinoma patients (*n* = 135) and 20.00% of SCC patients (*n* = 43). An overview of the histological regression score stratified by neoadjuvant therapy scheme is shown in Figure 1. An alluvial plot illustrating the flow from tumour entity to administered neoadjuvant therapy and resulting histopathological regression grade is provided in Figure 2.

### 3.3 Univariate Statistics

In univariate analyses, BMI did not differ significantly across histopathological tumour regression grades. As the assumption of normality was violated (Shapiro–Wilk test on ANOVA residuals, *p* < 0.001), a non‐parametric Kruskal–Wallis test was applied, which did not demonstrate a statistically significant global association between BMI and regression grade (*p* = 0.871). Nevertheless, descriptive comparisons suggested a tendency (*χ* ^2^ = 0.71, df = 3, *p* = 0.871) towards higher BMI values in patients with partial (grade 2; median 25.2 kg/m^2^) and subtotal regression (grade 1b; median 25.7 kg/m^2^) compared with those showing either complete regression (grade 1a; median 25.0 kg/m^2^) or minimal/no regression (grade 3; median 25.1 kg/m^2^). Pairwise Wilcoxon rank‐sum tests reflected this directional pattern, although none of the comparisons reached statistical significance (all unadjusted *p* > 0.40), and no pairwise differences remained significant after Bonferroni correction (all adjusted *p* = 1.0). Importantly, these patterns persisted after exclusion of underweight patients, while overall group differences remained non‐significant (Kruskal–Wallis *p* = 0.600).

### 3.4 Multivariate Statistics

After univariable analyses, a multivariable ordinal regression model was conducted including all prespecified covariates. Assessment of the proportional odds assumption was attempted by comparing proportional and partially non‐proportional specifications. BMI showed clear threshold‐dependent effects and was therefore modelled with non‐parallel (cut‐point–specific) coefficients in the final partial proportional odds model. To ensure model stability, covariates were dichotomised where appropriate. For age (kept linear)—which displayed borderline non‐parallel effects—allowing non‐proportional effects did not yield a meaningful improvement and was not retained. To improve model robustness, the proportional odds assumption was retained for binary predictors.

In the final partial proportional odds model, higher BMI was independently associated with lower odds of achieving unfavourable histopathological regression, with the magnitude of this association increasing at higher regression thresholds. Specifically, BMI did not significantly correlate with decreased odds ratios (OR) of achieving at least Becker regression grade 1b. However, higher BMI was associated with reduced odds of achieving Becker grade 2 (OR 0.97, 95% CI 0.94–1.00) and Becker grade 3 (OR 0.93, 95% CI 0.90–0.96), indicating a progressively stronger inverse association at higher levels of histopathological regression. Exploratory analyses of non‐linear BMI effects using quadratic and cubic terms did not provide stable or statistically meaningful improvements in model fit and were accompanied by numerical instability; BMI was therefore retained as a linear predictor with threshold‐specific (non‐parallel) effects. SCC histology, absence of dysphagia, RCTX as neoadjuvant treatment regimen and lower ECOG score were independently associated with lower odds of unfavourable regression, while no independent associations were observed for diabetes, comorbidity burden, age, sex or pretherapeutic weight loss. In particular, a sensitivity analysis excluding underweight patients yielded equivalent results: a higher BMI was associated with reduced odds of achieving favourable histopathological regression, with effects becoming progressively stronger at higher regression thresholds. Likewise, excluding cM1 cases, the BMI association remained virtually unchanged compared with the main model. Overall model fit of the main model was modest (McFadden's pseudo‐*R* ^2^ = 0.068). The results of the final multivariable model are summarized in Table 5.

**TABLE 5:** Adjusted odds ratios (OR), 95% confidence intervals (CIs) and variance influencing factor (VIF), from the final partial proportional odds model (cumulative logit). Reference categories in brackets.

Predictor	VIF	OR	95% CI	*p*
BMI (Becker score ≥ 1b vs. lower)	—	1.01	[0.98, 1.04]	0.58
BMI (Becker score ≥ 2 vs. lower)	—	0.97	[0.94, 1.00]	0.04*
BMI (Becker score ≥ 3 vs. lower)	—	0.93	[0.09, 0.96]	< 0.01**
Diabetes (yes vs. no)	1.06	1.28	[0.85, 1.94]	0.23
Histology: SCC (vs. adenocarcinoma)	1.78	0.55	[0.36, 0.82]	< 0.01**
Neoadjuvant: RCTX (vs. CTX)	1.70	1.61	[0.41, 0.89]	0.01*
Comorbidity (vs. no)	1.03	0.97	[0.72, 1.30]	0.83
Age (per year)	1.05	1.00	[0.98, 1.01]	0.64
Female sex (vs. male)	1.07	1.15	[0.80 1.66]	0.44
ECOG < 2 (vs ≥ 2)	1.04	1.66	[1.17, 2.35]	< 0.01**
No dysphagia (vs. yes)	1.02	1.64	[0.45, 0.89]	< 0.01**
No weight loss (vs. yes)	1.03	0.92	[0.66, 1.25]	0.58

*Note:* Significance levels: *p < 0.05, **p < 0.01.

In treatment‐modality‐stratified analyses, a higher BMI remained associated with lower odds of poor histopathological regression, particularly a Becker grade ≥ 3, in both the chemotherapy and radiochemotherapy subgroups. There was no evidence of divergent effect directions between the two. Furthermore, in an additional robustness analysis replacing the broad comorbidity variable with a registry‐adapted CCI and adding alcohol consumption, nicotine pack years and time since smoking cessation, the association with BMI remained stable. However, the supplementary variables showed no relevant univariate association with Becker regression and did not improve the overall model fit. Therefore, the original model was retained.

**FIGURE 3:**
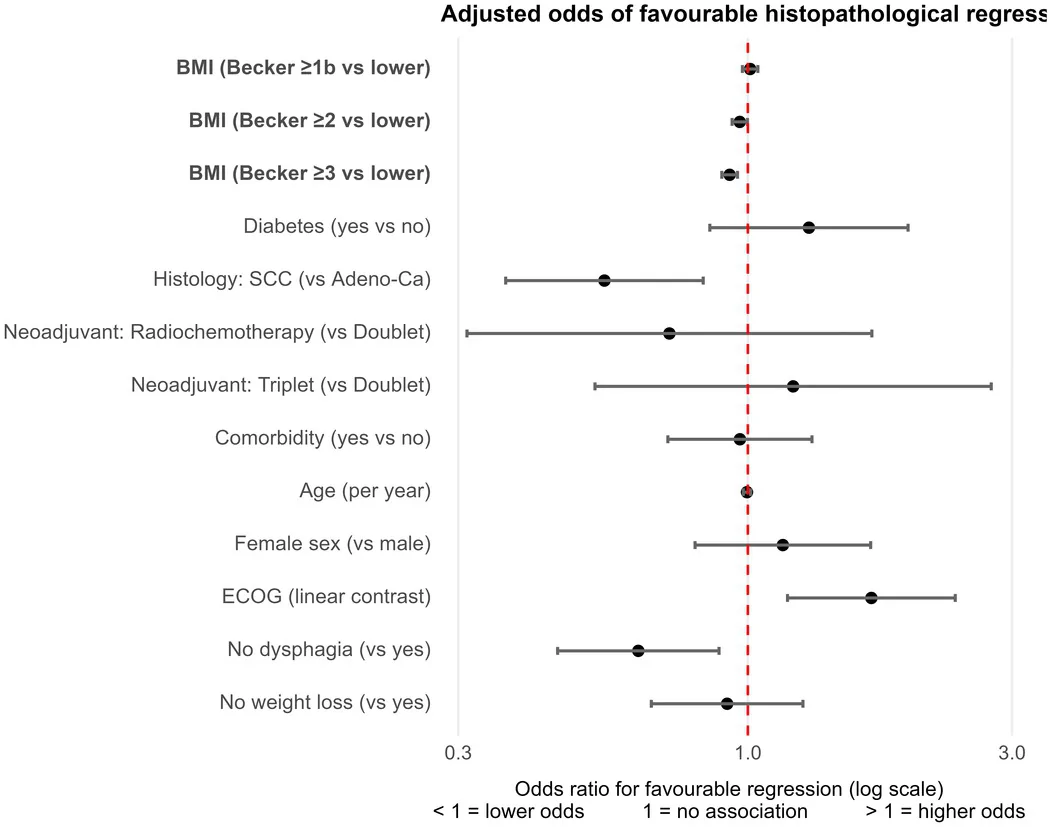
Forest plot of adjusted odds ratios from the partial proportional‐odds model for histopathological regression after neoadjuvant therapy. Increasing BMI is associated with reduced odds of unfavourable regression with increasingly pronounced effects in higher regression scores.

A graphical overview regarding the variables of influence is presented in Figure 3 as a forest plot.

## 4 Discussion

### 4.1 Key Findings

As obesity rates are rising worldwide, the knowledge of the influence of body weight on the outcome of cancer treatment becomes increasingly relevant. In this study, we investigated the impact of BMI as primary explanatory variable on tumour regression after neoadjuvant therapy for oesophageal cancer, using the prospective database from the German nationwide DGAV|registry of the DGAV. Multivariate regression analyses revealed a significant association between BMI and tumour regression score. After adjustment for clinical and tumour‐related covariates, higher BMI was independently associated with progressively lower odds of achieving higher Becker regression grades. No association was observed for the transition to Becker grade ≥ 1b; however, higher BMI was associated with reduced odds of achieving Becker grade ≥ 2 (OR 0.97, 95% CI 0.94–1.00, *p* = 0.04) and Becker grade ≥ 3 (OR 0.93, 95% CI 0.90–0.96, *p* < 0.001). Furthermore, we found a significant independent association between higher odds of a more favourable regression score and the covariables histology (SCC), absence of dysphagia, RCTX as neoadjuvant treatment, and ECOG status < 2.

### 4.2 Influence of BMI on Neoadjuvant Treatment Response

Despite the rising obesity rates, the knowledge of the impact of BMI on multimodal treatment remains limited, and the literature on its prognostic value is conflicted. On one hand, obesity is associated with an increased risk for cancer development, poor histopathological response and surgical complications. These effects are attributed to metabolic dysfunction and, with respect to surgery, to heightened technical complexity [26, 27]. On the other hand, a growing number of studies suggest that an elevated BMI may correlate with improved overall survival, frequently referred to as the ‘obesity paradox’ [28, 29]. Possible explanations are a change of tumour microenvironment and metabolite pathways (Section 4.5).

Consequently, the overall impact of obesity on treatment outcome is a subject of ongoing controversy. While obesity is generally recognized to be associated with a reduced overall survival in most cancers, such as breast cancer and colorectal cancer, data from other cancer entities, including lung cancer, indicate a potential survival benefit [30]. For oesophageal carcinoma specifically, comprehensive data remain scarce, and the prognostic relationship is less defined, with studies reporting reduced, equivalent or even superior survival rates in obese patients [26, 27, 31].

This underlines the lack of knowledge to the effect of BMI especially in the treatment of oesophageal carcinoma and emphasizes the need for more detailed analysis. To our knowledge, there is only one study analyzing the influence of obesity on pathologic complete response after chemoradiotherapy for oesophageal carcinoma, published in 2014: In a retrospective analysis, Wang et al. found no association of a higher BMI to complete response in a multivariant logistic regression model. However, they described a reduced risk of chemoradiation‐associated complications in patients with higher BMI [32].

In other tumour identities, obesity has been found to affect the response to neoadjuvant treatment. Less complications and reduced pathologic complete response after neoadjuvant treatment in obese patients have been described for breast cancer and colorectal cancer [33]. Regarding the reduced radiation side effects, Wang et al. [32] suggest that visceral fat may act as a natural tissue separation, protecting normal tissue from radiation. Our findings do not support this hypothesis. In contrast to this study and in accordance to our findings, increased pathologic complete response rates in obese breast cancer patients, who received full uncapped doses of neoadjuvant treatment, have been seen by Farr et al. [34]. They suggest reduced pathologic complete response and reduced toxicity in the previous studies may be caused by reduced doses. Furthermore, they assume an unintended dose accumulation in obese patients, since chemotherapy is considered less effective in adipose tissue and therefore may induce more impact in target organs [34, 35].

### 4.3 Influence of Covariables on Neoadjuvant Treatment Response

In our cohort, a significant independent association was observed between higher odds of a more favourable regression score and the covariables histology (SCC), absence of dysphagia, RCTX as neoadjuvant treatment and ECOG status < 2.

Regarding histology, SCC exhibits a higher complete tumour remission rate following multimodal therapy compared with adenocarcinoma. These findings are consistent with existing clinical data and are generally accepted to be driven by differences in both tumour biology and treatment concepts. Because SCC is predominantly managed with neoadjuvant RCTX, these two factors overlap. This observation aligns with the landmark CROSS trial, which established that patients with SCC achieve a substantially higher pathological complete response rate under neoadjuvant RCTX compared to those with adenocarcinoma [36, 37].

Regarding the absence of dysphagia as an independent predictor of a better response, we hypothesize that this clinical symptom indicates both a lower local tumour burden and a reduced risk of complications such as aspiration, malnutrition and consecutive treatment interruption. This might contribute to a better therapeutic response in these patients. Accordingly, a favourable ECOG status reflects the patient's physiological resilience and thus a reduced risk of complications and incomplete neoadjuvant treatment.

### 4.4 Strengths and Limitations

To our knowledge, this is the first study to demonstrate a significant association between higher BMI and improved histopathological tumour regression after neoadjuvant therapy for oesophageal cancer. One of the strengths of this study is the large nationwide prospective collected database of the DGAV|registry provided by the DGAV. We included 647 patients and performed extensive multivariate regression analysis with adjustment for associated factors and confounders, including tumour histology, neoadjuvant regimen, age, sex and ECOG performance status, which enabled a robust statistical analysis.

We are aware of the study's limitations. As a surgical dataset, it only captures patients who underwent operative resection. Furthermore, granular data regarding chemotherapy cycles and radiation dosing were unavailable, which limits our ability to precisely correlate BMI with immediate neoadjuvant treatment response. Given that the delivered treatment intensity represents a potential confounding factor in therapeutic response, this lack of detail must be factored into the interpretation of our findings.

Analysis of multi‐institutional data is always subject to confounding and heterogeneity, making interpretation challenging. Additionally, because the exact number of participating centres was not collected within this registry, we cannot fully account for potential centre‐specific variations. Regarding BMI, possible confounders may be side effects of neoadjuvant treatment, dysphagia, reduced general health condition or cachexia due to advanced tumour stages, all of which can lead to weight loss and consecutive reduced BMI in patients with an overall reduced prognosis. Furthermore, higher BMI may represent high muscle mass rather than adipositas in healthy patients. While dysphagia and general health condition were included in our analysis, specific markers of nutritional status, muscle mass and weight loss velocity were not captured. We cannot exclude that higher BMI may reflect muscle‐driven resilience rather than purely adipose‐driven changes. To minimize possible interference and bias, we adjusted our multivariate analysis to as many relevant clinical and tumour parameters as stability allowed (e.g., weight loss, dysphagia, ECOG status, see Section 2).

### 4.5 Hypothesis: Tumour Biology

Obesity is associated with chronic low‐grade systemic inflammation and metabolic dysregulation, which are linked to alterations in cellular and tumour biology, including changes in proliferation and apoptosis and may contribute to an altered response to neoadjuvant treatment.

Adipocytes, especially in abdominal visceral adipose tissue, are metabolically active, leading to the release of biologically active mediators and an overall pro‐inflammatory state [8]. At tissue level, this is leading to an elevation of inflammatory cytokine expression, which contributes to angiogenesis, chemotaxis and cell migration and consecutively to cancer progression [7]. In addition, obesity is associated with an increase of growth factors, like free insulin‐like growth factor‐1 (IGF‐1) and insulin, which induce cellular proliferation and inhibit apoptosis, promoting carcinogenesis [7]. Furthermore, obesity promotes dysregulation of adipokines, which are released by adipocytes and support the regulation of body weight and peripheral energy expenditure. Leptin, which is elevated in obese patients, can also cause increased proliferation and inhibition of apoptosis. The leptin antagonist adiponectin decreases with adiposity, leading to a reduction of its protective effects [26]. Other aspects are elevated steroid hormones and oestradiol levels in obese patients, which may contribute to cancer pathogenesis as well [38]. Obesity may impact the tumour microenvironment through the altered circulation of adipokines, growth factors and endocrine signals. Possible mechanism of an influence on response to neoadjuvant treatment in obese patients may be the changed interaction of these biological or further tumour driving factors on drug and metabolite pathways, amplified by a dysregulation of corresponding receptors in tumour cells. A potential impact of body weight on treatment results has been observed in other treatment regimes, too. In immunotherapy for solid tumours, a better outcome in obese patients has been seen and authors discuss an obesity‐linked T cells exhaustion, which may be reversed by immunotherapy or provide an increased number of targets for immunotherapy in the tumour microenvironment [39, 40].

In general, poorer treatment outcomes in obese patients might be expected. Instead, obesity is reported to be associated with reduced risk of neoadjuvant treatment toxicities in oesophageal carcinoma [32, 39] and in accordance with our own findings increased complete response has been seen in obese patients with breast carcinoma [34].

Obesity is generally considered to exert pro‐carcinogenic effects. Accordingly, we hypothesized that patient populations may differ with respect to underlying tumour biology. While obesity may increase the risk of developing cancer, tumour behaviour in patients with obesity may be less aggressive. An alternative hypothesis is that neoadjuvant treatment may counteract obesity‐associated biological effects, potentially attenuating their impact on tumour progression and thereby modifying treatment response. Further mechanistic and translational studies are required to explore these hypotheses.

## 5 Conclusion

In conclusion, this analysis of the German nationwide prospective DGAV|registry database demonstrates for the first time that higher BMI is significantly associated with improved histopathological tumour regression following neoadjuvant treatment for oesophageal cancer. The results were independent from other factors and tumour characteristics in multivariate regression analysis, like tumour histology, neoadjuvant regimen, age, sex, ECOG performance status and presence of dysphagia.

These findings suggest that BMI may act as an independent predictor of histopathological regression following neoadjuvant therapy in oesophageal cancer and point towards potential differences in tumour biology among patients with obesity; however, causality cannot be inferred from the present data and warrants further investigation.

## Author Contributions

**Susanne D. Otto:** conceptualization, methodology, writing – review and editing, writing – original draft, validation, visualization. **Ioannis Pozios:** conceptualization, methodology, writing – review and editing. **Wael Rayya:** writing – review and editing, methodology. **Christian H. W. Schineis:** writing – review and editing, conceptualization. **Swantje Malinka:** writing – review and editing. **Rahel M. Strobel:** writing – review and editing, visualization. **Jonas Staudacher:** writing – review and editing, methodology. **Christoph Treese:** writing – review and editing, methodology. **Tim Vilz:** writing – review and editing, supervision. **Pia Jäger:** conceptualization, methodology, data curation, formal analysis, validation, visualization, writing – original draft, writing – review and editing.

## Funding

The authors have nothing to report.

## Disclosure

Permission to Reproduce Material From Other Sources: Permission for the use of the data of the DGAV|registry oesophageal surgery provided by the German Society for General and Visceral Surgery (Deutsche Gesellschaft für Allgemein‐ und Viszeralchirurgie, DGAV) has been granted.

## Ethics Statement

The authors have nothing to report.

## Consent

All patients have given informed consent to the collection and analysis of their medical data and history in the DGAV|registry oesophageal surgery provided by the German Society for General and Visceral Surgery (Deutsche Gesellschaft für Allgemein‐ und Viszeralchirurgie, DGAV).

## Conflicts of Interest

The authors declare no conflicts of interest.

## Data Availability

The data that support the findings of this study are available from DGAV|registry oesophageal surgery provided by the DGAV. Restrictions apply to the availability of these data, which were used under license for this study. Data are available from the author(s) with the permission of DGAV|registry oesophageal surgery provided by the DGAV.
